# The inequality of health-income effect in employed workers in China: a longitudinal study from China Family Panel Studies

**DOI:** 10.1186/s12939-020-01211-6

**Published:** 2020-06-15

**Authors:** Mengxue Xie, Zhiyong Huang, Wenbin Zang

**Affiliations:** School of Public Administration, Southwestern University of Finance and Economics, 555 Liutai Avenue, Chengdu, 611130 Sichuan China

**Keywords:** Health-income effect, Income distribution, Panel quantile regression, Inequality

## Abstract

**Background:**

The relationship between health and income is an essential part of human capital research. The majority of current analyses using classical regression models show that health has a significant impact on income after controlling for the endogeneity of health due to the measurement error and reverse causality. Currently, the Chinese government implements various policies including health related policies to fiercely fight for the domestic poverty issues, and thus only estimating the average effect of health on income could underestimate the impact for low income population and will make policy makers neglect or not pay enough attention to the significant role of health in poverty alleviation. To study the effect of health on income for workers at different income quantiles, we apply the quantile regression method to a panel data from a Chinese household survey. Furthermore, we test the heterogeneity of this health-income effect for different subgroups of workers characterized by sex, registered residence, and residential area. Lastly, we provide an explanation on the possible mechanism of the health-income effect.

**Methods:**

This study uses data from four waves of the China Family Panel Studies (CPFS)- a biennial longitudinal study spanning from 2012 to 2018. The final data used in the regression analysis includes a balanced sample of 19,540 person-year observations aged between 18 to 70 years, with complete information of demographic and social economic status characteristics, job information, and health status of individuals. We use lagged self-reported health to control the potential endogeneity problem caused by reverse causality between health and income. Our identification on heterogenous treatment effects relies on panel quantile regressions, which generate more information than the commonly used mean regression method, and hopefully could reveal the effects of health on income for workers with income distributed at a wide range of quantiles. In addition, we compare the results derived from panel quantile regressions and mean regressions. Finally, we added interaction terms between health and other independent variables to recover the influence channel of health on income.

**Results:**

The regression estimates show that the effects of health on income are more pronounced for workers distributed on the lower ends of income spectrum, and the health-income effect decreases monotonically with the increase of income. The treatment effect is robust to alternative measures of health and seems to be more pronounced for females than males, for rural workers than their urban counterparts. Finally, we find that health not only directly affects worker’s income but also has different effects on income for different occupation cohorts.

**Conclusions:**

This study provides a different perspective on the impact of individual health status on income, uncovering the heterogeneous effects of health deterioration on income reduction for workers with different incomes by using panel data and rather advanced statistical techniques- panel quantile regressions. At present, the Chinese government is making every effort to solve the problem of poverty and our findings suggest public policies on health and income protections should emphasize different needs of workers with different incomes and special attention should be paid to low-income workers who are much more financially fragile to health deterioration than other income groups.

## Background

Health is one of essential components of human capital and a great deal of current research focus on health-related topics. Studies on the relationship between health and income have a long history in social science and nowadays most researchers agreed that workers with better health in general are paid higher, and the improvement of health status can also effectively improve wages [1–4]. Good health has a significant favourable influence on the income of workers both in developed and developing countries [5–8].

However, the early research on the health-income effect was criticized for the endogeneity caused by the interaction between health and income [9–11]. Thus, some researchers expand econometric techniques from Ordinary Least Square (OLS) to instrumental variable, linear fixed/random effect, matching methods and quantile regressions to solve the problems ([12–14]]. For instance, lagged health indicators from the previous period were used as valid proxies of current health status.

Due to the heterogeneity of workers’ income, the impact of health on income for workers at different income distribution might not be the same. Some researchers apply panel quantile regression to explore this heterogenous relationship between workers’ health and income. Kniesner et al. [15] used this method to test the relationship between the statistical value of life and different wage levels in the United States, finding that higher health risk would lead to a significant reduction of income, and this effect is consistent for different subsamples. Hsieh et al. [16] using the data of family dynamic panel study (PSFD) in Taiwan and find a significant positive correlation between workers’ health status and wage rate. The healthier workers are, the more substantial contribution to their wages. Kedir [17] using panel data of Ethiopian households from 1994 to 2000 and find that both height and body mass index had a significant positive correlation with wages.

There were also abundant research focusing on developing economies, including Brazil [18], Ghana [19], India [20], Indonesia [21] and South Korea [22]. In China, the relationship between health and income is widely explored, suggesting that health human capital has a positive and significant effect on the wages of workers [23–25]. Health capital is an essential factor affecting the income of workers in China; workers with better health status have higher income.

Researchers using data from China have also made efforts to address endogenous problems of health, by adopting instrument variable method, simultaneous equation, and lagged indices [26–28]. Using lagged self-rated health as basic health indicator, Zhang [29] found a significant positive correlation between health and current labour supply, as well as income for both urban and rural residents. Qin et al. [30] used the Heckman model to study the impact of health on migrant workers’ income, which showed that health significantly affected the working probability of working for migrant workers, resulting in significant increase in income.

Being the second largest economy in the world, a labour force of high-quality is indispensable in ensuring the sustainable development for China. Thereby studies on health in the labour market won its growing interests among researchers and policymakers, especially in the context of the increasing income gap among workers. What’ s more, the Chinese government implements various policies including health related policies and propose the Health Poverty Alleviation project to fiercely fight for the domestic poverty issues, which makes it more crucial to study and understand the influence of health on income for low-income workers. This study aims to address the following questions: (1) What is the impact of health on different income workers, especially on lower- and middle-income workers? (2) What are the advantages of panel quantile regression compared with classical regressions? Thus, we use lagged self-reported health to solve the endogeneity problem caused by reverse causality of health and income, and compare the estimates of the fixed-effect model and panel quantile regression model.

Our study is not only limited to the estimation of the average effect, but also able to reveal the health-income influence for workers at different income quantiles, especially for those who are distributed at the lower tail of income distribution. The study of the relationship between health and income of workers in different income cohort groups will help us understand the role of health, and the highlight of the income inequality due to health will support the government when issuing targeted policies for low-income workers.

## Method

In this study, we use data from four waves of the China Family Panel Studies (CPFS)- a biennial longitudinal study spanning from 2012 to 2018. Our working sample includes 19,540 person-year observations aged between 18 to 70 years, with complete information of demographic and social economic status characteristics, job information, and health status of individuals. We use lagged self-reported health to control the potential endogeneity problem caused by reverse causality between health and income. Our identification on heterogenous treatment effects relies on panel quantile regressions, which generate more information than the commonly used mean regression method, and hopefully could reveal the effects of health on income for workers with income distributed at a wide range of quantiles.

### Data

The data are from the China Family Panel Studies (CFPS). The CFPS is a high-quality biennial household longitudinal survey, which is collected by the China Social Sciences Research Centre (ISSS) at Peking University. The survey focuses on the individual economic and non-economic welfare of Chinese residents, including individual’s employment status, educational achievement, health-related behaviours, family relationships, migration status and etc. Considering the great regional differences in Chinese society, CFPS adopts the proportional probability sampling (PPS) with implicit stratification, multi-stage, multi-level and population proportionality. The cross-sectional response rates of surveys in 2012 and 2014 were 74.1 and 72.8% respectively, and the cross-survey tracking rates were 80.6 and 83.8% respectively. As for the missing data, CFPS team has adopted a variety of processing methods to reduce the missing rate. For example, if personal income is missing, replace it with the average of the income range. If it is still missing or less than 100 yuan, it shall be replaced by the summation of the sub items related to income(see [31], for more details about CFPS). CFPS has so far conducted six surveys from 2010 to 2018, and each survey covers 25 provinces and cities with a total sample of 16,000 households and approximately 30,000 family members in these households.

In this study, we use data from waves of 2012, 2014, 2016, and 2018. After dropping observations with missing information on health indicators and other covariates, the final sample of our study includes 19,540 person-year observations aged between 18 and 70 years.

### Variables

The key independent variable in our analysis is self-reported health. Compared with other health indicators such as healthcare use, disability, illness history and biomarkers [6, 32–34], self-reported health is less costly and easy to administer in large social surveys such as CFPS. In addition, self-reported health has been shown a good predictor of both mortality and morbidity and large use in literature [35, 36]. Besides, we use lagged self-reported health as a proxy of current health to deal with the endogeneity problem caused by the reverse causality between health and income.

Self-reported health are extracted from the survey questionnaire and measured on a Likert scale with levels of excellent, very good, good, average and poor. We merge excellent, very good and good into one category due to the rather limited sample size of individuals whose reported health are excellent and very good health.^1^

Table 1 provides the distribution of self-reported health in each wave. First, we observe that most people reported their health as good or above, accounting for 71% of observations. 24.4% reported their health as average and 4.6% as poor. Second, we can also detect a time change. In the year of 2010, only 2.6% of workers reported poor health, against 7.6% reporting poor in the year of 2016. In contrast, 78.1% of workers reported good health in 2010, but the proportion decreased to 64.6% in 2016. Table 1 suggests that the health status of workers was getting worse over the period of 2010 and 2016.

**Table 1:** Distribution of Self-Reported Health in Each Survey Wave

Self-reported health	In total	2010	2012	2014	2016
Good	0.710	0.781	0.741	0.671	0.646
Average	0.244	0.193	0.228	0.278	0.278
Poor	0.046	0.026	0.032	0.051	0.076

The outcome variable is the self-reported annual income from the individual’s current jobs, calculated as the sum of salary, various bonuses and allowances of workers. We choose annual income instead of monthly wages as the dependent variable because the former measure is less subject to monthly variation which might be common for certain type of jobs. It is worth mentioning that we exclude farmers or self-employed workers since they do not get paid by employers. The annual income is calculated in RMB Yuan in 2018 value. Table 2 below shows the average annual income of individuals with different self-reported health status in each wave and in all four waves.

**Table 2:** The Average Annual Income of Workers in Each Wave in RMB Yuan

Wage	2012	2014	2016	2018	Overall
Health					
Good	27,441	33,732	33,289	39,828	33,283
Average	24,886	25,417	26,994	33,335	28,018
Poor	21,428	21,781	23,943	24,163	23,308

The overall average annual income for workers who reported good, average, and poor health are 33,283 Yuan, 28,018 Yuan, and 23,308 Yuan, respectively. It suggests that workers with better self-reported health have higher earnings. Table 2 also shows that workers’ annual income increased continuously over the years from 2012 to 2018, but the income gap between different health cohorts became larger overtime. This tells us better health not only brings a higher earing for workers in China but also causes the income gap to widen overtime. Though the descriptive analysis in Table 2 strongly suggests a correlation between health and income, this correlation can be a spurious relationship without further control on other potential confounding factors.

Another main independent variable is worker’s occupation type. We divide workers into two groups, formally and informally employed workers. The former includes workers employed by the government, public and private enterprises, and large-scale organizations with labour contract. The latter includes workers who do not have a formal labour contract or a long-term contractual relationship with their employers. This distinction is important since workers of unstable employment were more vulnerable to shocks such as health deterioration. A dummy indicator is used to represent an individual’s occupation, with one for workers who were formally employed and zero otherwise.

In regressions, we control a rich set of characteristics of workers including educational attainment, age and its squared term, sex, nationality, registered residence as urban and rural, marital status as well as residential area^2^ as East China, Central China, Northeast China and West China.

### Estimation models

We apply a Mincer [37] equation in which the log of annual income is modelled as a function of education and experiment in human capital. The general form of the Mincer Equation as follows:

1 $$ \mathit{\ln}(y)=f\left( Sch, Exper,X,\varepsilon \right) $$

Where *ln*(*y*) denotes the log of income, *Sch* and *Exper* denote the schooling and working years. *X* represents the other independent variables than education and experience, including sex, marital, living areas. Other unobservable factors are contained in the error term *ε*,which satisfied *E*(*ε*| *X*) = 0.

Also, the effect of health on productive time and thereby income is hypothesized in Grossman model [3], which add new a perspective of the human capital research that health is a component that affects income. Following the expansion of Mincer Equation in previous literature, we model the log of annual income as a function of health status, age, sex, education, marital status, and other of covariates. Endogeneity may arise if unobserved individual characteristics are correlated with health variable. In panel data, by assuming that these unobserved individual characteristics as time-constant, we can use fixed effect models. The model presents as in Eq. (2):

2 $$ {lnincome}_{it}={\alpha}_i+{H}_{it}^{\prime}\upbeta +{X}_{it}^{\prime}\gamma +{\varepsilon}_{it} $$

Where the subscripts *i* and *t* denote individual and survey year, respectively. β and *γ* are estimated parameters. *lnincome*_*it*_ denotes the log of the annual income of individual *i* measured in RMB Yuan in time *t*, *H*_*it*_ denotes health status of individual *i* in time *t*. *X*_*it*_ is a series of dummy indicators of an individual’s characteristics as covariates. *α*_*i*_ represents the individual specific effect, which is assumed time-irrelevant in models of fixed effects. *ε*_*it*_ is a random error term and is often assumed to be iid normally distributed.

The majority of current analyses using classical regression models show that, on average, health has a significant impact on income. However, in the case of the widening income gap, estimating the average effect of health on income would underestimate the impact for poor workers and underrate the inequality of the health-income effect. To better understand the heterogeneous treatment effects of health on income for individuals with varying income levels, we resort to quantile regression.

Quantile regression was first introduced by Koenker and Bassett in 1978 [38], as an extension of the classical regression model and the panel quantile regression is quantile regression using panel data. Considered a linear model of the τ th quantile.

$$ {y}_i={x}_i^T{\beta}_{\tau }+{e}_i,\mathrm{i}=1,\dots, \mathrm{n}, $$,

Where the τ th quantile of *e*_*i*_ is zero. The estimator of *β*_*τ*_ can be derived from solving:

$$ \hat{\beta_{\tau }}= argmin\sum \limits_{i=1}^n{\rho}_{\tau}\left({y}_i-{x}_i^T\beta \right) $$

The quantile regressions are modelled as the conditional quantiles of the dependent variable and solved by minimizing the sum of weighted residual absolute values of residuals instead of minimizes the sum of squared residuals in OLS estimates. Compared with linear models, the quantile models are often less biased to skewed data and could capture varying effects. Therefore, quantile regressions fit non-normal distributed data and result in more robust estimates.

In addition, quantile regressions are well-known to be more robust to the outliers and require much weaker assumptions for the distribution of the error term compared with classical linear regressions.

The panel data quantile regression is the extension of quantile regression in panel data by Koenker [39]. Considered Eq. (2) where the τ th quantile of the conditional distribution of *lnincome*_*it*_ given explanatory variable vector *X* is specified as:

3 $$ {Q}_{\tau}\left(\mathrm{l}{nwage}_{it}\ |\ X\right)={\alpha}_i+{X}^T\beta \left(\tau \right),\uptau \in \left(0,1\right). $$

Where *Q*_*τ*_(*lnwage*_*it*_ | *X*) denotes the quantile τ of log monthly wage conditional on the vector of regressors.

The panel quantile model combines the advantages of panel data and quantile regression, in the sense that it effectively controls unobserved individual heterogeneity while being more robust to functional misspecification. Using panel quantile regression, we are able to recover the relationship between the dependent variable and other covariant based on the distribution of dependent variable at different quantiles [40]. The results of panel quantile regression show the different effects for specific quantile with robustness, which is suitable for our research.

In addition, we are also interested in the heterogeneity of the health-income effect among different demographic and socio-economic groups measured as sex, register residence and residential area. As male and female workers are endowed with different physical and mental capacity, they are expected to have different levels of health and income [41, 42]. According to the existing literature, there are vast differences between rural and urban residents in China, with the effect of health on wages is more pronounced for rural residents [43, 44]. Moreover, we test the health impact based on residential area.

Finally, we analyse the influence mechanism of health-income effect, and discuss the influence path of health on income by adding the interaction terms between health and other variables.

Finally, we analyse the influence mechanism of health-income effect, and discuss the influence path of health on income by adding the interaction terms between health and other variables.

Concerned with the problem of multicollinearity in regressions, we report bootstrap errors (bootstrap of 1000 times) rather than the asymptotic standard error to guarantee the efficiency of the regression model. All statistical and regression analysis is performed using R 3.5.1.

## Results

### Descriptive analyses

The descriptive statistics for both the dependent variable and independent variables are presented in Table 3.

**Table 3:** Descriptive Statistics

Variables	Mean (Standard Deviation) [Minimum, Maximum]		
	ALL(N = 19,540)	Male(N = 11,604)	Female(N = 7936)
*Annual Income* (RMB Yuan)	31,538(28,547) [2300/200,000]	34,226 (30,181) [2300/200,000]	27,607 (25,465) [2300/200,000]
*Self-reported health*			
Good	0.710	0.697	0.728
Average	0.244	0.256	0.227
Poor	0.046	0.047	0.045
*Age*	45.8 (10.1) [18,70]	46.4 (10.5) [18,70]	44.8 (9.4) [18,70]
*Education*			
Primary and below	0.355	0.313	0.417
Junior high	0.362	0.390	0.321
Senior high or College	0.237	0.256	0.210
University and above	0.046	0.041	0.052
*Occupation*	0.377	0.421	0.375
*Registered residence*	0.695	0.688	0.704
*Married*	0.703	0.705	0.701
*Nationality (Han)*	0.936	0.938	0.934
*Residence Area*			
East China	0.370	0.369	0.371
Central China	0.236	0.244	0.224
Northwest China	0.370	0.369	0.371
West China	0.168	0.167	0.170

The overall average annual income is 31,538 Yuan, with 34,226 Yuan for males and 27,607 Yuan for females. 69.7% males reported good health, and the proportion for female is 72.8%. On average, female workers are 2 years younger than male workers. More than two thirds of males (70.3%) and females (73.7%) have completed education lower than senior high school, while 29.7% of males and 26.3% females have completed higher education. Approximately 68.8% of males and 70.4% of females live in rural areas and about 45.1% males and 39.5% females have formally employed works.

### Regression estimates

The first column of Table 4 shows the regression result using the fixed-effect model, (2) to (5) columns show the panel quantile regression results at 25th, 50th, 75th, and 90th quantile of the income distribution.

**Table 4:** Regression Results of FE and Panel Quantile Regression

	Fixed-Effect	Panel Quantile Regression			
		(2) 25th	(3) 50th	(4) 75th	(5) 90th
Good health	0.158^∗ ∗ ∗^ (0.020)	0.219^∗ ∗ ∗^ (0.071)	0.169^∗ ∗ ∗^ (0.053)	0.164^∗^ (0.085)	−0.064 (0.093)
Average health	0.113^∗ ∗ ∗^ (0.019)	0.171^∗∗^ (0.070)	0.123^∗∗^ (0.052)	0.082 (0.087)	−0.131 (0.095)
Age	0.035^∗ ∗ ∗^ (0.002)	−0.008^∗ ∗ ∗^ (0.001)	−0.012^∗ ∗ ∗^ (0.001)	−0.010^∗ ∗ ∗^ (0.001)	−0.006^∗ ∗ ∗^ (0.001)
Sex	–	0.179^∗ ∗ ∗^ (0.012)	0.267^∗ ∗ ∗^ (0.014)	0.209^∗ ∗ ∗^ (0.015)	0.116^∗ ∗ ∗^ (0.015)
Marital	0.044 (0.010)	0.167^∗ ∗ ∗^ (0.013)	0.285^∗ ∗ ∗^ (0.015)	0.307^∗ ∗ ∗^ (0.016)	0.215^∗ ∗ ∗^ (0.019)
Registered residence	−0.399^∗ ∗ ∗^ (0.020)	−0.290^∗ ∗ ∗^ (0.017)	−0.225^∗ ∗ ∗^ (0.019)	−0.070^∗ ∗ ∗^ (0.018)	−0.005 (0.019)
Junior high	–	0.061^∗ ∗ ∗^ (0.014)	0.093^∗ ∗ ∗^ (0.018)	0.050^∗ ∗ ∗^ (0.018)	0.012 (0.017)
Senior high or College	–	0.149^∗ ∗ ∗^ (0.017)	0.210^∗ ∗ ∗^ (0.021)	0.150^∗ ∗ ∗^ (0.022)	0.089^∗ ∗ ∗^ (0.021)
University and above	–	0.485^∗ ∗ ∗^ (0.033)	0.520^∗ ∗ ∗^ (0.036)	0.403^∗ ∗ ∗^ (0.040)	0.404^∗ ∗ ∗^ (0.072)
Occupation	0.227^∗ ∗ ∗^ (0.009)	0.450^∗ ∗ ∗^ (0.018)	0.397^∗ ∗ ∗^ (0.022)	0.446^∗ ∗ ∗^ (0.027)	0.675^∗ ∗ ∗^ (0.029)
Central China	–	−0.142^∗ ∗ ∗^ (0.016)	−0.184^∗ ∗ ∗^ (0.022)	−0.120^∗ ∗ ∗^ (0.019)	−0.109^∗ ∗ ∗^ (0.021)
Northeast China	–	−0.182^∗ ∗ ∗^ (0.020)	−0.277^∗ ∗ ∗^ (0.022)	−0.274^∗ ∗ ∗^ (0.022)	−0.229^∗ ∗ ∗^ (0.025)
West China	–	−0.196^∗ ∗ ∗^ (0.024)	−0.287^∗ ∗ ∗^ (0.027)	−0.293^∗ ∗ ∗^ (0.027)	−0.266^∗ ∗ ∗^ (0.027)

^a^ Numbers in parentheses are estimated robust standard errors corrected for clustering using bootstrap technique

^b^ ***, **, * Significance levels at 1, 5, and 10% respectively

^c^ Poor health as the reference group

The fixed-effect model suggests that, the workers with average and good health have an average annual income 15.8 and 11.3% higher than ones of poor health, after controlling for age, marital status, registered residence, and occupation. Due to the specification of the fixed-effect model, time-irrelevant variables such as sex, education, residential areas are dropped.

Apart from this mean effect recovered by linear fixed effect models, panel quantile regressions indicate that workers reporting their health as good and average earned 21.9 and 17.1% higher than those who report poor health at lower income levels (e.g. the 25th quantile). The gaps narrow down to 16.9 and 12.3% for the median income group and further to 16.4 and 8.2% at higher income levels (e.g. the 75th quantile). These estimates suggest that workers distributed at the 25th and 50th percentile of income are affected more by health and health status may have an insignificant effect on workers at the upper tail of the income distribution. Other variables have signs as expected. For example, education is statistically significant at conventional levels at all quantiles, which is consistent with existing empirical evidence on the return of schooling.

Furthermore, we have conducted the falsification test which include the forward treatment variable as additional controls – a way commonly used as the post estimation test in panel data regressions – to test the model assumptions of our analysis. In the test regressions, we have found that when the health in prior period is controlled, the current health is only marginally significant in fixed effect model, and mostly insignificant in panel quantile regression, which supports our identification assumption. The test results are shown in the Table 11 in Appendix.

**Fig. 1:**
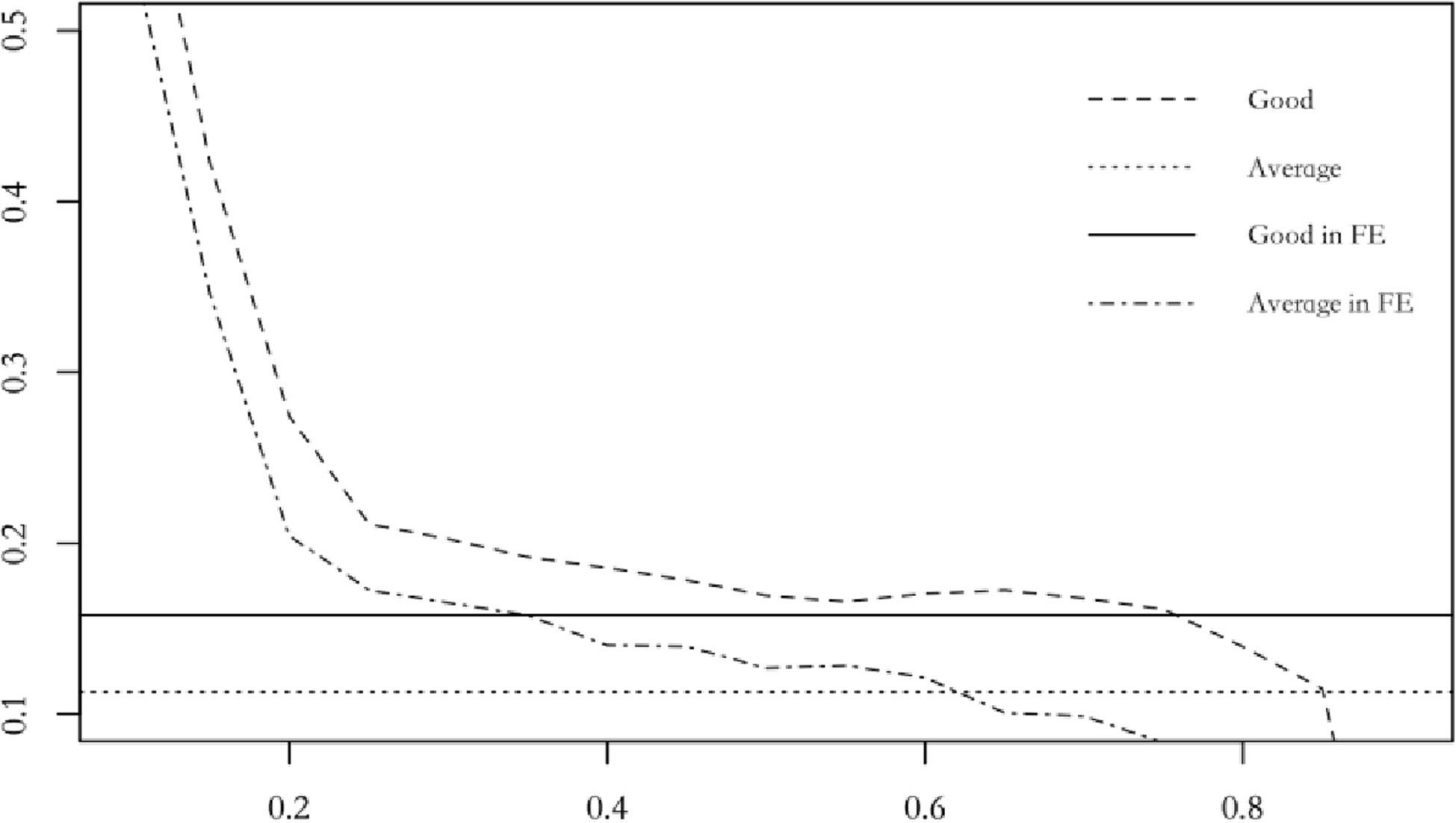
Comparisons of the Fixed-Effect Estimates and Panel Quantile Regression Estimates

Figure 1 depicts effects of health on annual income on the full range of income quantiles from zero to one. The dashed lines represent the coefficient estimates for have the average and good health against bad health using the panel quantile model and the solid line and dotted line represent the coefficient estimates using the fixed-effect model. It is evident that the lower- and middle-income workers are more affected by health status.

From Fig. 1 we can conclude that, first, better health brings higher income in the sense that good or average earn more than those reporting poor. Second, the effect of health on income dampens with the increase of the income distribution, suggesting a more crucial health-income effect for low- income workers. Third, the fixed-effect model estimates have roughly the same magnitude as panel quantile estimates at 75th quantile, suggesting that the classical estimation may underestimates the health-income effect for low-income workers but overestimates the effect for high-income workers. On the other hand, using panel quantile regression, we are able to recover heterogeneous returns of health on income regarding different income groups while controlling of endogeneity.

### Sensitivity analyses

In this section, we discuss the relationship between health and income, using alternative health indicators to verify the reliability of our previous finding. Following Cai et al. [45], we generate a new health index called ‘health score’, which combines two questions ‘Have you suffered from a chronic or acute disease?’ and ‘How severs was the illness or injury?’ in the questionnaire. Based on responses to the two questions, health score is defined as an ordered response variable with four different levels: no chronic disease (s1), have a chronic illness but not severe (s2), have chronic diseases somewhat severe (s3) and quite severe (s4). We treat health score as a binary variable with four levels.

Table 5 shows the estimation results of the panel quantile regression model using health score as the health indicator, which suggests better health measured as health score dummies significantly implies better earnings for those whose income are distributed at 25th and 50th quantile, after controlling for covariates. Nevertheless, the effect is insignificant for workers whose income are distributed at the upper ends.

**Table 5:** Panel Quantile Regression Results Using Health Score Variable

	Panel Quantile Regression Estimation			
	25th	50th	75th	90th
Score 1	0.262^∗ ∗ ∗^ (0.054)	0.183^∗ ∗ ∗^ (0.041)	0.221^∗ ∗ ∗^ (0.056)	0.086 (0.073)
Score 2	0.235^∗ ∗ ∗^ (0.057)	0.148^∗ ∗ ∗^ (0.045)	0.155^∗∗^ (0.063)	0.003 (0.077)
Score 3	0.171^∗ ∗ ∗^ (0.054)	0.133^∗ ∗ ∗^ (0.043)	0.119^∗∗^ (0.060)	−0.023 (0.077)
Controlling Covariates	YES	YES	YES	YES

^a^ Numbers in parentheses are estimated robust standard errors corrected for clustering using bootstrap technique

^b^ ***, **, * Significance levels at 1, 5, and 10% respectively

^c^ All estimations control for the following covariates: sex; education; age and its squared term; registered residence; marital status; nationality, dummy indicators for the residential area

^d^ Health score is defined as an ordered response variable with four different levels: no chronic disease (s1), have a chronic illness but not severe (s2), have chronic diseases somewhat severe(s3) and quite severe(s4). s4 is used as reference group.

Compared with panel quantile regression using lagged self-rated health, the results in column (2) to (5) in Table 4 and Table 5 are similar, which indicates that our regression results are robust to particular health measures used.

### Heterogeneity analysis

Table 4 shows that male workers’ incomes are higher than females at all quantiles after controlling for other factors, but the pattern is not clear. To test the potential differences in the effect of health on income for sex heterogeneity, we discuss the health-income effect by sex.

Table 6 shows the health-income effects by sex using the self-reported health index. Estimations reveal that health is more important for female workers than males, since the health-income effect is more significant for females. Male workers with good and average health earned slightly higher annual income than male workers with poor health, but it is not statistically significant for most male workers. As the distribution annual income increased, the health-income effect shows a downward trend. For instance, annual income for males with average health is 13.4% higher than those with poor health at 25th quantile, but only 7.5% higher and insignificant at 75th quantile.

**Table 6:** Panel Quantile Estimated Health-Income Effect by Sexes

		25th	50th	75th	90th	Obs.
Male	Good	0.134^∗^ (0.076)	0.145^∗∗^ (0.068)	0.075 (0.113)	−0.163 (0.135)	11,604
	Average	0.093 (0.075)	0.086 (0.065)	−0.028 (0.114)	−0.143 (0.127)	
Female	Good	0.413^∗∗^ (0.191)	0.291^∗∗^ (0.083)	0.270^∗ ∗ ∗^ (0.104)	0.121 (0.131)	7936
	Average	0.366^∗^ (0.190)	0.276^∗∗^ (0.083)	0.210^∗∗^ (0.126)	0.057 (0.13)	

^a^ Numbers in parentheses are estimated robust standard errors corrected for clustering using bootstrap technique

^b^ ***, **, * Significance levels at 1, 5, and 10% respectively

^c^ All estimations control for the following covariates: education; age and its squared term; registered residence; marital status; nationality, dummy indicators for the residential area

For female workers, the health-income effects are larger for workers placed at lower and middle range of the income distribution than those placed at the higher end. The similar pattern is observable for males even though with smaller effects. For example, females with good health earn 41.3 and 29.1% higher than those with poor health at the 25th and 50th percentiles, but only 12.1% higher at the 90th quantile and no longer significant.

**Fig. 2:**
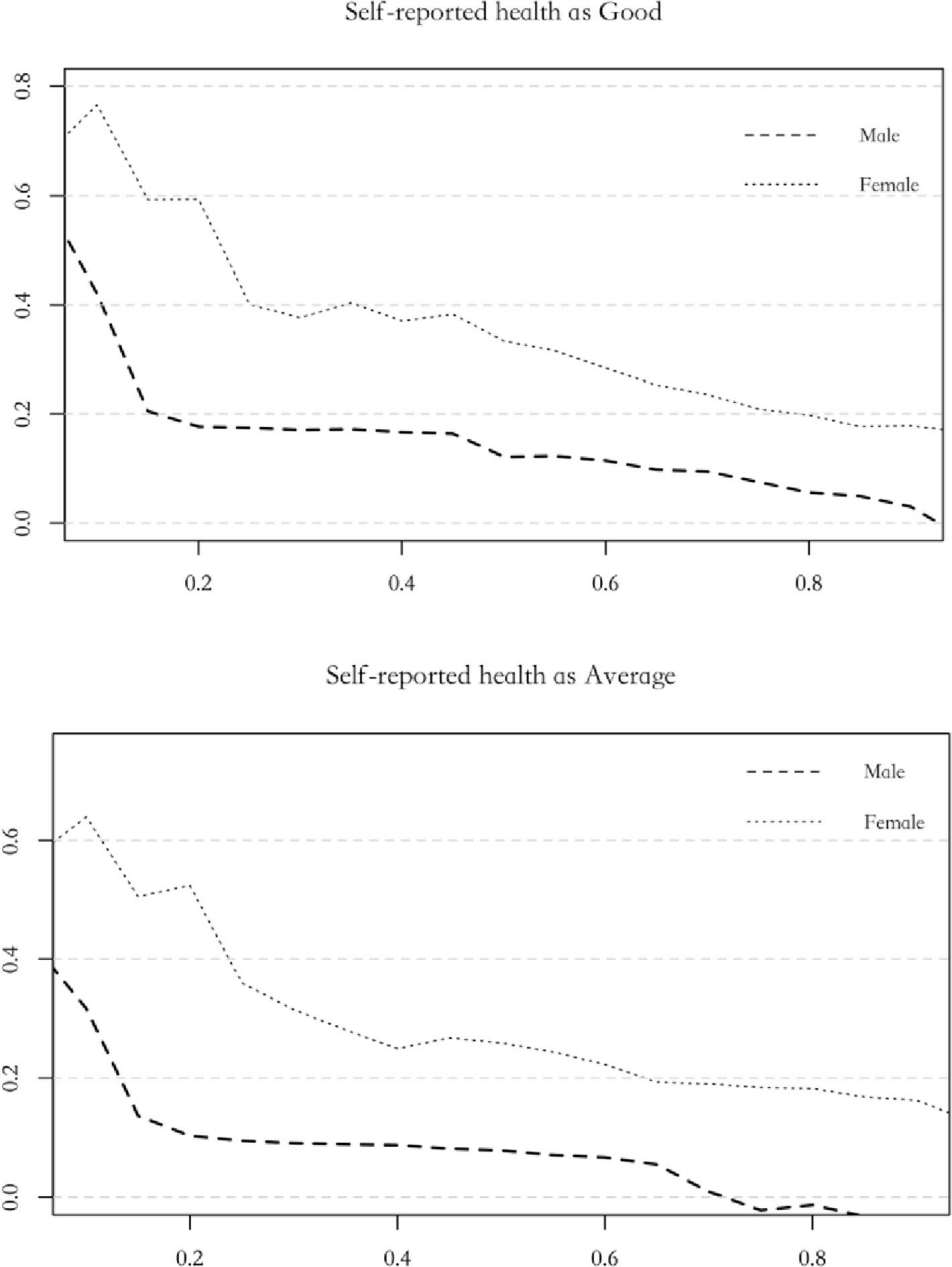
Panel quantile estimates by sexes under different health level

Figure 2. shows the variation of panel quantile estimates for workers with different sex and health status under full income distribution. The dashed line represents male and dotted line represents female. We find that female workers are more affected by health than males for two health statuses. With the increase of income distribution, the health-income effect for females decreasing faster than males, which means health is more important for female workers than males, especially for low-income workers.

Next, we test the heterogeneity of the registered residence for workers. In general, rural workers earned 39.9% less than urban workers on average in the fixed-effect model, but in the panel quantile regression, the income gap is relatively small. Even for rural workers that distributed at 25th quantile, the income gap is 29%, and the difference is narrowing along with the annual income distribution. So, we are interested in how did health affect income for workers with the different registered residence.

From the regression results in Table 7, the effect of rural workers’ health on income is higher than that of urban workers. For urban workers, the impact of health on income is not significant for all income quantiles. For rural workers, health has a considerable effect on income for most workers, and the health-income effect is decreasing of the income distribution, from 25.5 to 17.5%, but is insignificant at 90th income distribution. Generally speaking, health is vital for rural workers, maintain good health contribute to higher income.

**Table 7:** Panel Quantile Estimated Health-Income Effect by Registered Residence

		25th	50th	75th	90th	Obs.
Urban	Good	0.105 (0.092)	−0.013 (0.119)	−0.067 (0.159)	−0.296 (0.213)	5964
	Average	0.075 (0.094)	−0.062 (0.122)	−0.137 (0.162)	−0.281 (0.216)	
Rural	Good	0.255^∗∗^ (0.106)	0.238^∗∗^ (0.112)	0.175^∗ ∗ ∗^ (0.071)	−0.018 (0.100)	13,576
	Average	0.197^∗^ (0.103)	0.161^∗^ (0.094)	0.109^∗∗^ (0.048)	−0.070 (0.102)	

^a^ Numbers in parentheses are estimated robust standard errors corrected for clustering using bootstrap technique

^b^ ***, **, * Significance levels at 1, 5, and 10% respectively

^c^ All estimations control for the following covariates: sex; education; age and its squared term; marital status; nationality, dummy indicators for theresidential area

The last heterogeneity test in this paper is the health-income effect for workers from different residential areas. Since we will assess whether the achievements of economic growth were distributed across the country, we divide these 25 provinces and cities into four economic regions as East China, Central China, Northeast China, and West China. Among these four economic regions, the East has the most powerful economic strength, followed by the Central and Northeast, and West is the weakest.

We present the quantile regression results grouped by four economic regions in Table 8 below to show the variance effect of health on income across the country. Table 8 shows four economic regions have the different health-income effect, and the effect is more significant for the relatively developed area in east China.

**Table 8:** Health-Income Effect in Four Economic Regions

	25th	50th	75th	90th	Obs.
East China	0.298^∗∗^ (0.150)	0.268^∗∗^ (0.117)	0.182^∗^ (0.115)	−0.091 (0.235)	7232
Northeast China	0.189 (0.211)	0.179 (0.124)	0.065 (0.148)	−0.027 (0.146)	3284
Central China	0.269^∗^ (0.164)	0.170^∗^ (0.162)	0.164 (0.114)	0.050 (0.182)	4608
West China	0.210^∗ ∗ ∗^ (0.080)	0.180^∗ ∗ ∗^ (0.072)	0.166 (0.170)	−0.085 (0.285)	4416

a Numbers in parentheses are estimated robust standard errors corrected for clustering using bootstrap technique.

b ***, **, * Significance levels at 1, 5, and 10% respectively.

c All estimations control for covariates: sex; education; age and its squared term; registered residence; marital status; nationality.

### Mechanism analysis

From previous regressions, we find that the health-income effects are robust even when we used different health indicators and consider the heterogeneity of workers. The observed health-income effect weakens with the increase of income of workers and are more relevant to low- and middle-income workers. Our hypothesis for the results is that health not only directly affects workers’ income but also affects income through workers’ occupation choices. Given the segmented job market in China, we have recognized the financial implication of health change would be quite different for workers with more secured jobs like those belonging to public sector and workers with less secured jobs. We hypothesized that the secured jobs would help to buffer the negative effect of health deterioration on income reduction. To verify our hypothesis, we have included an analysis in which occupation is dichotomized as stable and unstable jobs and interacted with health. Thus, we divide occupations in the questionnaire into two categories based on job characters. The first category, termed as formal employment, consists of jobs with long-term contracts with employers such as enterprise and government and regular working hours and salaries but with requiring higher education or job skills. The other category that we term as informal employment include workers who engage on manual or repetitive work with lower requirements of education or professional knowledge, who are temporarily employed or paid by days and easily replaced.

The income distributions of these two types of workers are illustrated in Table 9. We find that with the increase of the income quantile, the proportion of workers engaged in formal employment was higher, while low-income workers were more involved in informal employment. The percentage of the formal employment increased from 8.7% at the lower tail of income to 68.8% at the upper.

**Table 9:** Descriptive Statistics of Occupation Categories by Income Quantiles

Quantile	Informal employment	Formal employment	Pct. of Formal employment	Annual Income
0–25%	4465	424	0.087	9368
25–50%	3102	1782	0.365	18,576
50–75%	3086	1796	0.463	30,126
75–100%	1523	3362	0.688	68,095
Obs.	12,176	7364	0.377	31,538

We add the interaction of health and occupation in the fixed-effect model and the panel quantile regression to explain how health affects income. We also apply mean-centering method [46] to reduce nonessential collinearity problem after including interaction terms. The results are presented in Table 10.

**Table 10:** Mechanism Analysis of Health-Income Effect

	Fixed-effect Model		Panel Quantile Regression			
	(1)	(2)	25th	50th	75th	90th
Good	0.158^∗ ∗ ∗^ (0.044)	0.128^∗ ∗ ∗^ (0.022)	0.219^∗ ∗ ∗^ (0.076)	0.134^∗∗^ (0.054)	0.144 (0.110)	−0.074 (0.096)
Average	0.113^∗ ∗ ∗^ (0.038)	0.085^∗ ∗ ∗^ (0.021)	0.168^∗∗^ (0.074)	0.093^∗^ (0.054)	0.061 (0.111)	−0.146 (0.099)
Occupation	0.227^∗ ∗ ∗^ (0.026)	0.229^∗ ∗ ∗^ (0.009)	0.458^∗ ∗ ∗^ (0.018)	0.298^∗ ∗ ∗^ (0.022)	0.469^∗ ∗ ∗^ (0.028)	0.678^∗ ∗ ∗^ (0.029)
Good*Job	–	−0.140^∗ ∗ ∗^ (0.042)	−0.183^∗∗^ (0.092)	−0.102^∗^ (0.055)	−0.087 (0.206)	−0.103 (0.144)
Average*Job	–	−0.127^∗ ∗ ∗^ (0.043)	−0.124^∗^ (0.071)	−0.048 (0.090)	−0.099 (0.208)	−0.144 (0.156)
Controlling Covariates	YES	YES	YES	YES	YES	YES

^a^ Numbers in parentheses are estimated robust standard errors corrected for clustering using bootstrap technique

^b^ ***, **, * Significance levels at 1, 5, and 10% respectively

^c^ Other covariates like gender, education, nationality and residential area are neglected in the fixed-effect model, and controlled in panel quantile regression

In both the fixed-effect model and the panel quantile model, the interaction between health and occupation is significantly and negatively correlated with income, that the health-income effect may only hold for workers with unstable employment. For those with stable employment, the employment serves as the safety net which could buffer the shock of health deterioration on income decrease. These results are highly suggestive that health not only affects income directly but also affects income through workers’ occupation.

## Discussion

Using the data of China Family Panel Studies from 2012 to 2018, we explore the relationship between health and income on the whole range of income distribution by panel quantile regressions. We first use lagged self-reported health as the health indicator to solve the endogenous problem caused by reverse causality between health and income, and then examine the robustness of our findings by using an alternative health measure calculated as a score of health conditions. Next, we test the heterogeneity effect of health on income for workers grouped by sex, registered residence, and residential areas to investigate heterogeneous treatment effects for each subgroup. At last, we test the potential mechanism how health affects income through occupational status.

Generally, we find that health has a significantly positive effect on income, but this effect varies with incomes. The return to health is higher for the lower- and middle- income workers than higher-income workers, which suggests the existence of health-income effect inequality for workers. Healthy workers earned an average 15.8 and 11.3% more than poor health workers according to the fixed-effect model, but these average estimates could potentially underestimate the health-income gap for the low- and middle- income workers and fail to uncover the inequality among labour forces. By using panel quantile regression, we note that the impact of health on income is more significant for low- and middle- income workers. In addition, we find that the influence of health on income dampens as income increase.

Some previous studies have considered the varying effect of health on income using income quartile to reduce the impact of income heterogeneity [47, 48]. However, we show that panel quantile regression method could generate a more comprehensive profile on the relationship between health and income, and could shed more lights on the topic, as suggested by the results of Table 4 and Fig. 1. For example, in Table 4, we find the income effect of workers with good health distributed at the 50th and 75th income quantiles are 16.9 and 16.4%, respectively, with a decrease of only 0.5%. On the other hand, the effect of health on income for workers distributed between 50th and 75th quantile has experienced growing first, then declining and then rising again as suggested in Fig. 1, which could not be obtained only by income group analysis.

Considering the complexity and availability of health measures, we adopt the health score index as an alternative health indicator in the panel quantile regression. The results show that for low- and middle- income workers, one point gained in health score resulting 6.1 and 5.2% higher wages, which is similar to the results of lagged self-rated health.

Table 6 presents that health-income effect is more crucial for female workers, which is consistent with some findings in the literature showing that the contribution of health on income was higher for women than men [33, 49]. One possible explanation for the difference in the health-income effect is the distinct social roles assumed by males and females. Compared with female workers, male workers, who are often expected to undertake more economic responsibility for the family and the society, wouldn’t leave the labour market even if their health status deteriorates. In contrast, female workers might reduce their labour supply or even quit the job and return to family in the case of health deterioration. Therefore, given equal levels of health, female workers are more motivated to join the labour market for higher income. Besides, female workers are more sensitive to the impact of diseases and medical insurance, which makes them more valuable in family health production [24].

The results present in Table 7 saying that rural workers have a higher health-income effect support the findings in previous literature that health status is more important for rural workers in China [50, 51]. Due to the restrictions of living regions, educational background, job skills, among other factors, most of the rural workers can only be engaged in manual jobs that relies heavily on health conditions.

Health status not only directly affects the income of the workers but also affects the income through the types of occupations. We find that workers who participated in manual labour were more dependent on their health than those involved in non-manual works. Therefore, the workers engaged in the unstable income occupation are more sensitive to the change of health status. As the workers involved in the unstable income occupation gradually decrease with the distribution of income, resulting in a reduced health-income effect.

Our findings have substantial policy implications. First of all, the government should increase public investment on maintaining and improving workers’ health, which is a cost-effective way to build sustainable development on human capital of the working population. Secondly, the government should focus on improving the health security level of low-income workers to reduce inequality, especially for female and rural workers. Low-income workers were characterized by worse health condition and lower disposable income, making them vulnerable to health risks such as illnesses and injuries. Two types of policies targeting on lower- and middle-income workers can be considered by the government: the first to increase health capital investments for workers with lower income, for example, by providing health care access and health care knowledge. The second is to increase the medical insurance benefits for the lower- and middle-income workers, thereby improving their health risk resistance.

### Strengths and limitations

To the best of our knowledge, no previous study has explored inequality of health-income effect for workers distributed on a wide income spectrum in China using panel quantile regression. In addition to the discussion of the health-income effect, we have investigated its heterogeneity for different demographic and socio-economic groups and possible mechanism to explain the effect. Our main finding that deterioration of health most affect workers located at the lower tail of income distribution, suggests that public policies should be tailored particularly to alleviate the negative impact of health deterioration on income for lower and middle-income groups.

This study has some limitations. First, the lagged self-reported health, which is used as the primary health measure in our study to deal with the endogeneity problem, may suffer from the measurement error in the sense that it is self-reported and lagged. To reduce the self-report bias, we have examined an alternative measure which is based on chronic illness history and hopefully less subject to self-report bias. Using lagged measure of health might be problematic given that health is a continuous and changeable indicator. Generally speaking, lagged health is often better than current health. When we use lagged health as an exposure variable to analyse the impact of health on income, the effect can be overestimated. Nevertheless, in our analysis, we have found that the income gap exists not only between workers who are placed at the two ends of health spectrum – the ones with good health and with bad health, but also exists between the ones with poor health and with average health. This speaks that despite the possible overestimation, the true effect of health-income gradient is unlikely to be neglectable. Second, to avoid the temporal variation we measure income of workers by their annual income, which, however, may be subject to year-to-year variation. Nevertheless, it is well-known that measurement errors for the outcome variable can only result in less efficiency of parameter estimates, but not bias. Finally, we have only examined occupation as the possible mediator accounting for the health-income effect. Even that we have found that occupation is indeed one important channel, there might be other important channels, which we have to leave for future research.

## Conclusions

This study provides new knowledge on the impact of health change on income reduction. First, this study reveals the heterogeneous effects of health deterioration on income reduction for workers with different incomes by using unique panel data and rather advanced statistical techniques – panel quantile regressions. The main finding is that workers with lower incomes are more likely to be affected by worsening health. Second, how this health-income effect differs among subgroups defined by urban/rural residence and geographic residence has been examined. Finally, mechanism of health affecting income through mediators such as occupation has been investigated. Findings of the current study confirms the necessity of social safety net on hedging the risk of income reduction due to health shocks. In particular, public policies on health and income protections should emphasize different needs of workers with different incomes and special focus should be given to low-income workers who are much more financially fragile to health deterioration than other income groups.

## Data Availability

All data came from public domain and could be accessed from the link below: 1) China Family Panel Survey: http://isss.pku.edu.cn/cfps/download/login [Original data, register and login to download both English and Chinese version]. 2) Mendeley Data repository: 10.17632/p8ddwcyhnv.1 [The datasets generated and analysed during the study].

## Appendix 1

**Table 11:** Placebo test on asumptions of model identification

	FE	FE panel quantile regression			
		(2) 25th	(3) 50th	(4) 75th	(5) 90th
Good health in time t-1	0.167^∗ ∗ ∗^ (0.020)	0.192^∗ ∗ ∗^ (0.053)	0.155^∗ ∗ ∗^ (0.039)	0.153^∗ ∗ ∗^ (0.060)	−0.102 (0.091)
Average health in time t-1	0.125^∗ ∗ ∗^ (0.019)	0.142^∗ ∗ ∗^ (0.052)	0.094^∗ ∗ ∗^ (0.037)	0.064 (0.059)	−0.173 (0.192)
Good health in time t	−0.030^∗^ (0.017)	0.016 (0.023)	0.010 (0.032)	0.039 (0.028)	0.088 (0.061)
Average health in time t	−0.036^∗∗^ (0.015)	0.046^∗∗^ (0.022)	0.042 (0.030)	0.070 (0.045)	0.103 (0.128)
Age	0.035^∗ ∗ ∗^ (0.002)	−0.008^∗ ∗ ∗^ (0.001)	−0.012^∗ ∗ ∗^ (0.001)	−0.010^∗ ∗ ∗^ (0.001)	−0.006^∗ ∗ ∗^ (0.001)
Gender	–	0.185^∗ ∗ ∗^ (0.016)	0.263^∗ ∗ ∗^ (0.014)	0.225^∗ ∗ ∗^ (0.015)	0.119^∗ ∗ ∗^ (0.021)
Marital	0.045^∗ ∗ ∗^ (0.010)	0.182^∗ ∗ ∗^ (0.011)	0.282^∗ ∗ ∗^ (0.012)	0.314^∗ ∗ ∗^ (0.016)	0.220^∗ ∗ ∗^ (0.018)
Living regions	−0.399^∗ ∗ ∗^ (0.020)	−0.263^∗ ∗ ∗^ (0.020)	−0.209^∗ ∗ ∗^ (0.024)	−0.050^∗ ∗ ∗^ (0.016)	0.018 (0.019)
Junior high	–	0.045^∗∗^ (0.020)	0.081^∗ ∗ ∗^ (0.026)	0.035^∗ ∗ ∗^ (0.016)	0.017 (0.016)
Senior high or College	–	0.158^∗ ∗ ∗^ (0.025)	0.219^∗ ∗ ∗^ (0.030)	0.141^∗ ∗ ∗^ (0.028)	0.092^∗ ∗ ∗^ (0.030)
University and above	–	0.519^∗ ∗ ∗^ (0.041)	0.560^∗ ∗ ∗^ (0.046)	0.409^∗ ∗ ∗^ (0.054)	0.358^∗ ∗ ∗^ (0.063)
Occupation	0.227^∗ ∗ ∗^ (0.009)	0.443^∗ ∗ ∗^ (0.018)	0.285^∗ ∗ ∗^ (0.018)	0.265^∗ ∗ ∗^ (0.020)	0.165^∗ ∗ ∗^ (0.023)
Co-variates	YES	YES	YES	YES	YES

^a^Numbers in parentheses are estimated robust standard errors corrected for clustering using bootstrap technique

^b^***, **, * Significance levels at 1, 5, and 10% respectively

## Footnotes

1 The percentage of workers who reported their health as “excellent” and “very good” is 5.3 and 8.8%, respectively. The effect of aggregated sample is 2.4% understated compared to the separated results. We believe it is acceptable and will not bias the results.

2 Beijing, Tianjin, Hebei, Shanghai, Jiangsu, Zhejiang, Fujian, Shandong, Guangdong are East China. Shanxi, Anhui, Jiangxi, Henan, Hubei, Hunan, are Central China. Liaoning, Jilin, Heilongjiang are Northeast China, Guangxi, Chongqing, Sichuan, Guizhou, Yunnan, Shanxi, Gansu are West China.
